# Case Report: Innate Immune System Challenge Unleashes Paraneoplastic Neurological Autoimmunity

**DOI:** 10.3389/fneur.2020.598894

**Published:** 2020-12-10

**Authors:** Mingqin Zhu, Yuetao Ma, Anastasia Zekeridou, Vanda A. Lennon

**Affiliations:** ^1^Department of Laboratory Medicine and Pathology, Mayo Clinic, Rochester, MN, United States; ^2^Department of Neurology, Mayo Clinic, Rochester, MN, United States; ^3^Department of Immunology, Mayo Clinic, Rochester, MN, United States

**Keywords:** neural autoantibodies, paraneoplastic autoimmune neurological disorders, PAMPS, onconeural peptides, cancer immunotherapy

## Abstract

Paraneoplastic autoimmune neurological disorders reflect tumor-initiated immune responses against onconeural antigens. Symptoms and signs can affect the central and/or peripheral nervous systems, neuromuscular junction or muscle, and typically evolve subacutely before an underlying neoplasm is discovered. We describe four patients whose neurological symptoms were precipitated by potent innate immune system challenges: bladder instillation of BCG, tick bite and an “alternative cancer therapy” with bacterial extracts and TNF-α. We hypothesize that a tumor-initiated autoimmune response (evidenced by autoantibody profiles), pre-dating the immune system challenge, was unmasked or amplified in these patients by cytokines released systemically from innate immune cells activated by microbial pathogen-associated molecular patterns (PAMPs). The resultant upregulation of cognate onconeural peptides as MHC1 protein complexes on neural cell surfaces would render those cells susceptible to killing by CD8+ T cells, thus precipitating the patient's neurological symptoms.

## Introduction

Paraneoplastic neurological autoimmunity is recognized most frequently with small-cell lung cancer (SCLC), thymoma, breast carcinoma, and ovarian or testicular neoplasms (1). Neural autoantibodies found in the patient's serum or cerebrospinal fluid (CSF) are products of B cells activated by immunogens released from tumor cells and co-stimulated by antigen-specific helper T cells (2, 3). These IgGs do not necessarily predict a specific neurological syndrome (4, 5), nor are they neuropathogenic, unless reactive with the extracellular domain of a plasma membrane autoantigen. Autoantibodies of cytoplasmic and nuclear specificities are surrogate markers of cytotoxic CD8^+^ T cell activation (6).

As serological biomarkers, neural-specific autoantibodies aid early cancer diagnosis. Each one generally associates with a restricted number of cancers, e.g., collapsin response-mediator protein 5 (CRMP5)-IgG and anti-neuronal nuclear antibody type 1 (ANNA-1)-IgG associate with SCLC (more rarely thymoma or neuroblastoma), Purkinje cytoplasmic antibody type 1 (PCA-1)-IgG with mullerian or breast carcinoma, Ma2-IgG or Kelch-like Protein 11 (KLH11)-IgG with seminoma (7), and N-methyl-D-aspartic acid receptor (NMDAR)-IgG with ovarian teratoma (3). Neurological symptoms sometimes appear years before cancer detection, and the triggering factors are largely unknown. In a service diagnostic autoimmune serology laboratory, we encountered four cases whose neurological symptoms were apparently precipitated or amplified by a potent systemic challenge to the innate immune system.

## Case Presentations

Case 1: A 68-year-old man, with a >20 pack years smoking history, completed intravesical Bacillus Calmette-Guerin (BCG) therapy for recurrence of *in situ* urothelial cancer involving bladder and prostatic urethra. Non-productive cough, malaise and chills occurred 1 month later, soon after a tick-bite. Oral doxycycline was prescribed by the primary care physician who suspected Lyme disease. In the following months, progressive cerebellar ataxia evolved. CSF at onset was inflammatory (Table 1). Brain MRI was normal. Despite negative Lyme serology, intravenous ceftriaxone was given empirically for 6 weeks. Neurological symptoms worsened and he lost 40 pounds. Lyme serology and CSF PCR testing (repeated at Mayo Clinic 4 months after onset) were negative, but CRMP5-IgG was found (serum titer, 240; normal value <240). Evaluation for cancer recurrence revealed a left kidney mass; nephrectomy identified foci of disseminated urothelial carcinoma. The patient stabilized neurologically post-surgery, without immunotherapy. The diagnosis was cerebellitis. He had no evidence of cancer for 5 years. Four cutaneous cancers were diagnosed and treated within the next 2 years: 3 carcinomas (basal cell, dorsum; squamous cell, leg; basal cell, pinna) and melanoma *in situ* (chest). At year 8, CRMP5-IgG titer was 15,360; chest CT showed calcified pre-tracheal lymph nodes and right lung granuloma, but no tumor. This serological finding was consistent with an active anti-tumor immune response and suggested an underlying SCLC. He survived until age 91.

**Table 1:** Patients whose paraneoplastic neurological autoimmunity was precipitated or exacerbated by innate immune system challenge.

**Patient No. Sex/Age (yrs)**	**Cancer**	**Challenge**	**Neurological symptoms**	**Interval (weeks after challenge)**	**Neural IgG auto antibodies detected**	**CSF findings**	**Tobacco use (pack years)**	**Immunotherapy**	**Follow-up years (status)**
1. M/68	Bladder and prostate (urothelial); skin (basal cell, squamous cell, melanoma)	BCG; tick bite	Cerebellar ataxia	4	CRMP5	13 WBC (88% lymphocytes), protein 80 mg/dL, OCB positive	20	None	24 (died 91 y.o.)
2. M/72	Bladder (urothelial)	BCG	Sensorimotor neuropathy, chorea	2	ANNA-1, CRMP5	Normal	22	IVMP, cyclophos-phamide	2 (alive)
3. F/39	Ovary	Extracts of “bacteria, thymus, spleen and liver”; TNF-α	Cerebellar ataxia	1	PCA-1	Not available	0	Plasmapheresis,	2 (alive)
4. F/74	Carcinoid (metastasis)	Tick bite, Babesia	Encephalopathy	3	VGKC, VGCC-P/Q, VGCC-N, CRMP5, GAD65, AChR, Striational	7 WBC (88% lymphocytes), protein normal, no OCB	0	IVMP	7 (dead)

*ANNA-1, anti-neuronal nuclear antibody, type 1 (aka “anti-Hu”); BCG, bacillus Calmette-Guerin; Ca, carcinoma; CRMP5, collapsin response-mediator protein-5; GAD65, glutamic acid decarboxylase-65 kDa; IVMP, intravenous methylprednisolone; AChR, nicotinic muscle acetylcholine receptor; OCB, supernumerary oligoclonal bands; PCA-1, purkinje cell cytoplasmic antibody, type 1 (aka “anti-Yo”); TNF-α, tumor necrosis factor-α; VGCC, voltage-gated calcium channel; VGKC, voltage-gated (Kv1) potassium channel-complex (not subtyped for LGI1 or CASPR2 specificities); WBC, white blood cells*.

Case 2: A 72-year-old man, with 22 pack years smoking history, presented soon after bladder urothelial carcinoma resection with left arm neuropathic pain accompanied by numbness and weakness. Within 2–3 weeks of intravesical BCG treatment, symptoms worsened and spread to the right arm and leg. EMG showed axonal asymmetric sensorimotor neuropathy, consistent with mononeuritis multiplex. Sural nerve pathology demonstrated reduced myelinated fiber density, multifocal axonal degeneration and epineurial mononuclear inflammatory infiltrates. Symptoms stabilized with steroid therapy. By 7 months, the patient had lost 20 pounds despite good appetite; generalized asymmetric chorea was diagnosed (a classical manifestation of paraneoplastic CRMP5 autoimmunity (8). Brain and spinal cord MRIs, CSF, and metabolic and toxicological studies were unremarkable. At month 8, autoimmune serology revealed two neural autoantibodies that strongly predict SCLC, ANNA-1-IgG, and CRMP5-IgG. PET/CT did not demonstrate cancer recurrence but wedge excision of a FDG-PET hypermetabolic pulmonary nodule revealed necrotizing granulomas that were negative for acid fast bacilli. Neurological symptoms stabilized but did not improve after cyclophosphamide therapy. Two years later no cancer was detectable.

Case 3: A 39-year-old woman, non-smoker, had a 4 year history of locally invasive ovarian carcinoma with multiple recurrences. Serum CA-125 remained persistently elevated despite surgery, chemotherapy, hormonal therapy and radiotherapy. She sought “alternative cancer immunotherapy” with intravenous immune globulin, tumor necrosis factor alpha (TNF-α) and “purified extracts of bacteria, thymus, spleen and liver.” Fever and subacute cerebellar ataxia developed during her treatment. Six months later she was wheel-chair-confined by ataxia with no evidence of tumor progression or brain metastasis. PCA-1/anti-Yo IgG (consistent with neurological autoimmunity related to mullerian or breast carcinoma; 3) was detected in serum (titer: 61,440; normal value <240). Symptoms stabilized but did not reverse after plasmapheresis and one cycle of doxorubicin. A neck metastasis and neurological deterioration were recorded 2 years later.

Case 4: A 74-year-old woman, non-smoker with cardiovascular risk factors, presented with intermittent fever, chills, arthralgia, myalgia and anorexia after a tick bite. Peripheral blood smear confirmed babesiosis. After antibiotic treatment, severe encephalopathy with delirium developed. EEG did not show any seizure activity. Brain MR angiogram and MRI demonstrated no significant abnormalities. CSF was non-inflammatory and lacked evidence of current babesiosis or Lyme disease. Metabolic studies were unremarkable. The neural autoantibody profile in her serum was highly SCLC-predictive: voltage-gated calcium channel (both P/Q-type and N-type), voltage-gated (Kv1) potassium channel-complex, CRMP5, glutamic acid decarboxylase-65, muscle acetylcholine receptor, and sarcomeric (striational) specificities were positive (3). The family history was positive for lung carcinoma (brother), but full body PET/CT imaging and mammography were negative for malignancy. She improved dramatically following high dose i.v. methylprednisolone therapy and autoantibody levels fell. The therapeutic response was consistent with IgG-mediated autoimmunity targeting neural synapses. Six years later metastatic hepatic carcinoid tumor was diagnosed. She died the following year.

## Discussion

We describe 4 patients with cancer, evident at neurological diagnosis or later, in whom symptoms of paraneoplastic neurological autoimmunity began or were exacerbated following extraordinary challenges to the innate immune system. We propose that in each of these patients a preexisting anti-tumor immune response was unmasked by large-dose exposure to pathogen-associated molecular patterns (PAMPs) in live microbial products. PAMPs activate Toll-like receptors on innate immune cells, triggering systemic release of cytokines and chemokines (9).

**Figure 1:**
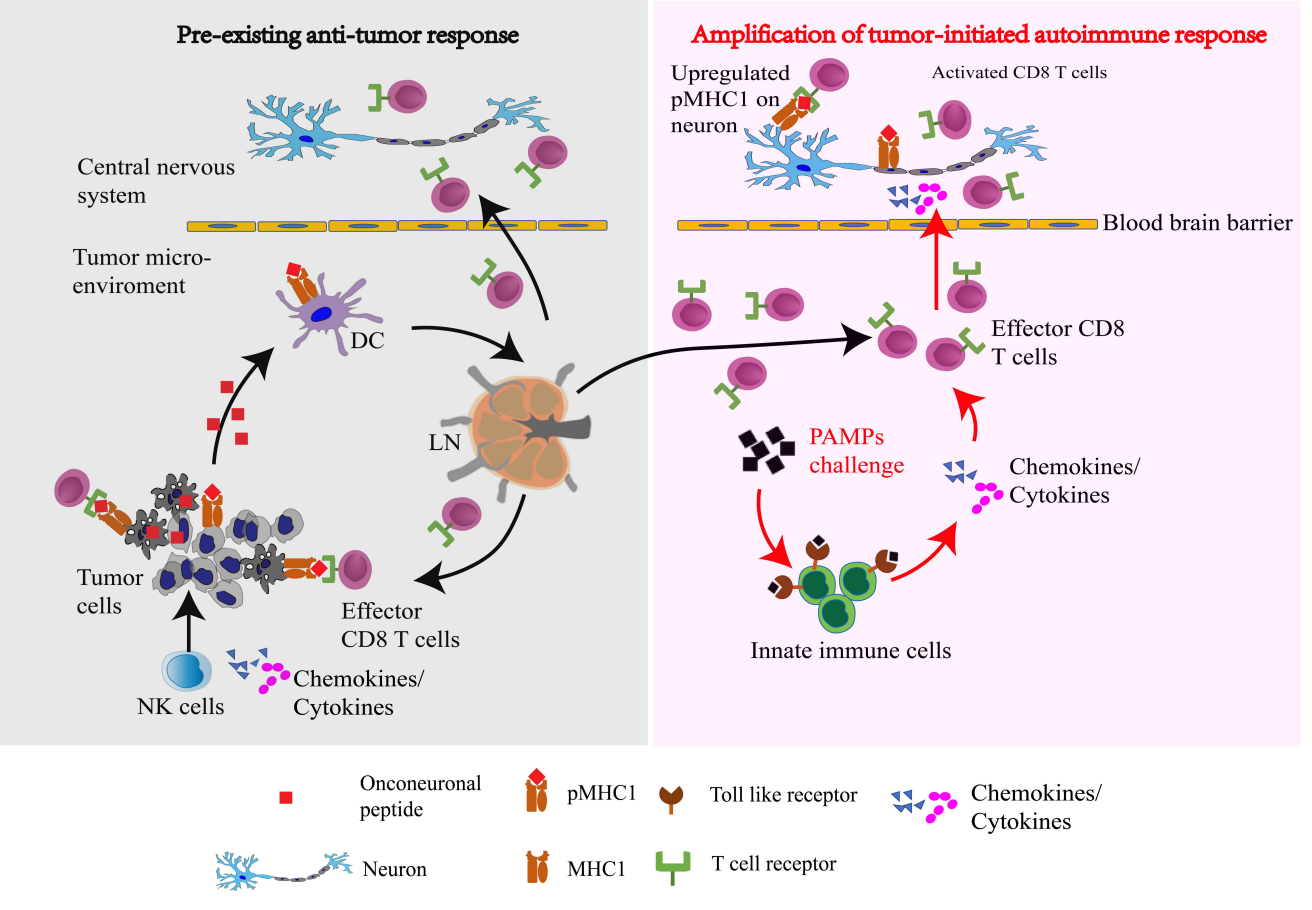
Proposed scheme by which pathogen-associated molecular patterns in a microbial challenge amplify, via systemically released cytokines, a pre-existing anti-tumor immune response to unleash a paraneoplastic neurological autoimmune response: Natural killer (NK) cells, the innate cytolytic immune responders to a nascent tumor, release onconeural peptides to dendritic cells which initiate an adaptive response in tumor-draining lymph nodes (LN). Intratumoral NK secretion of IFNγ and TNFα upregulates on the tumor cell surface the display of MHC class I (MHC1) proteins complexed with onconeural peptides (pMHC1) from the intracellular compartment. Antigen-primed CD8^+^ T cells emanating from lymph nodes circulate systemically and kill tumor cells by T cell receptor (TCR) engagement with tumor surface pMHC1 complexes. In response to large-dose challenge with microbial products, pathogen-associated molecular patterns (PAMPs) amplify systemic cytokine and chemokine release from innate immune cells via activation of their Toll-like receptors (TLRs). The resulting pro-inflammatory milieu promotes MHC1 upregulation on neural cells, rendering them susceptible to attack by activated CD8^+^ T cells entering the central nervous system, thus precipitating the patient's neurological presentation.

Onconeural proteins are normally restricted to neural cells, but when expressed in cancer cells they have potential to initiate paraneoplastic autoimmunity (1, 3, 6). Attack by natural killer (NK) cells, the “first responders” at the nascent tumor site, releases these antigens locally to dendritic cells, initiating an adaptive response in tumor-draining lymph-nodes (10). NK-released interferon (IFN)-γ and TNF-α at the tumor site would upregulate cancer cell surface display of MHC class I (MHC1) proteins complexed with onconeural peptides (pMHC1). Peptide-activated CD8^+^ T cells emerging from lymph nodes to circulate through all tissues would kill tumor cells by TCR engagement with tumor surface pMHC1 complexes (10). Despite activation of an autoimmune effector response, neurological symptoms are lacking or limited because surface MHC1 expression is minimal on quiescent nervous system parenchymal cells. However, in face of large-dose microbial product challenge, such as BCG inoculation (11) or tick bite (12), pro-inflammatory cytokines and chemokines are released by innate immune cells. The pro-inflammatory milieu elicited by systemically released cytokines and chemokines, could promote MHC1 upregulation on neural cells, rendering them susceptible to CD8^+^ T cell attack, and accounting for the patient's neurological presentation (Figure 1).

The efficacy of anti-tumor immune responses in patients with paraneoplastic autoimmunity is attested to by the survival of patient #1 for 24 years, despite development of 5 independent malignancies, and of patient #3 beyond 6 years, despite multiple cancer recurrences.

Both of this report's patients with urothelial carcinoma were abusers of tobacco products, which are a major cause of bladder cancer and the leading cause of SCLC. Although no lung carcinoma was found in the follow-up period, the autoantibody profiles in both patients were highly predictive of SCLC (3). It is estimated that lung cancer eventually will be diagnosed in 8% of men who have bladder cancer diagnosis (13). Our laboratory has observed that SCLC diagnosis is eventually made, usually limited and sometimes only at autopsy, in 13% of patients who have a SCLC-predictive autoantibody profile with initial detection of an unrelated cancer (14). It is a reasonable assumption that the malignant carcinoid eventually found in patient #4 (non-smoker, with SCLC-predictive autoantibody profile) expressed neuroendocrine antigens common to SCLC (15).

## Conclusions

We consider the unmasking of a pre-existing but silent autoimmune response through immune activation mediated by PAMPs to be a generalizable explanation for precipitation of paraneoplastic neurological autoimmune manifestations by microbial encounters, and perhaps more plausible than the notion of microbial “molecular mimicry” of neural antigens.

## Data Availability Statement

The original contributions presented in the study are included in the article, further inquiries can be directed to the corresponding author/s.

## Ethics Statement

All patients/subjects provided written informed consent for their medical records to be used for research purposes. However, no written informed consent was obtained specifically for the publication, as this was not required at the time patients were studied, more than 1 decade ago. The study carried out was reviewed and approved by the Mayo Clinic's institutional review board, including the consent procedures applied.

## Author Contributions

MZ: analysis and interpretation of data and revised the manuscript. YM: acquisition, analysis, interpretation of data, and initial manuscript draft. AZ: reviewed clinical history details and critically revised the manuscript. VL: study concept and design, acquisition, interpretation of data, and critically revised and edited manuscript. All authors contributed to the article and approved the submitted version.

## Conflict of Interest

AZ has a patent for PDE10A-IgG as a biomarker of paraneoplastic neurological autoimmunity. VL shares in royalties derived from Mayo Clinic licensing of commercial aquaporin-4 autoantibody testing. The remaining authors declare that the research was conducted in the absence of any commercial or financial relationships that could be construed as a potential conflict of interest.

## References

[1] Zekeridou A Lennon VA. Neurologic Autoimmunity in the Era of Checkpoint Inhibitor Cancer Immunotherapy. Mayo Clin Proc. (2019) 94:1865–78. 10.1016/j.mayocp.2019.02.003 31358366

[2] Albert ML Darnell RB. Paraneoplastic neurological degenerations: keys to tumour immunity. Nat Rev Cancer. (2004) 4:36–44. 10.1038/nrc1255 14708025

[3] Iorio R Lennon VA. Neural antigen-specific autoimmune disorders. Immunol Rev. (2012) 248:104–21. 10.1111/j.1600-065X.2012.01144.x 22725957

[4] Pittock SJ Kryzer TJ Lennon VA. Paraneoplastic antibodies coexist and predict cancer, not neurological syndrome. Ann Neurol. (2004) 56:715–9. 10.1002/ana.20269 15468074

[5] Horta ES Lennon VA Lachance DH Jenkins SM Smith CY McKeon A. Neural autoantibody clusters aid diagnosis of cancer. Clin Cancer Res. (2014) 20:3862–9. 10.1158/1078-0432.CCR-14-0652 24833664

[6] Albert ML Darnell JC Bender A Francisco LM Bhardwaj N Darnell RB. Tumor-specific killer cells in paraneoplastic cerebellar degeneration. Nat Med. (1998) 4:1321–4. 10.1038/3315 9809559

[7] Mandel-Brehm C Dubey D Kryzer TJ O'Donovan BD Tran B Vazquez SE. Kelch-like protein 11 antibodies in seminoma-associated paraneoplastic encephalitis. N Engl J Med. (2019) 381:47–54. 10.1056/NEJMoa1816721 31269365 PMC6800027

[8] Vernino S Tuite P Adler CH Meschia JF Boeve BF Boasberg P. Paraneoplastic chorea associated with CRMP-5 neuronal antibody and lung carcinoma. Ann Neurol. (2002) 51:625–30. 10.1002/ana.10178 12112110

[9] Mogensen TH Pathogen recognition and inflammatory signaling in innate immune defenses. Clin Microbiol Rev. (2009) 22:240–73, Table of Contents. 10.1128/CMR.00046-08 PMC2668232 19366914

[10] Schreiber RD Old LJ Smyth MJ. Cancer immunoediting: integrating immunity's roles in cancer suppression and promotion. Science. (2011) 331:1565–70. 10.1126/science.1203486 21436444

[11] Corral-Fernandez NE Cortez-Espinosa N Salgado-Bustamante M Romano-Moreno S Medellin-Garibay SE Solis-Rodriguez M. Induction of transcription factors, miRNAs and cytokines involved in T lymphocyte differentiation in BCG-vaccinated subjects. Mol Immunol. (2016) 77:44–51. 10.1016/j.molimm.2016.07.006 27454344

[12] Tappe D Booken N Boer-Auer A Rauch J Schmiedel S Reich K. Histology and Serum Cytokine Responses in an Imported Rickettsia slovaca Infection, Germany. Am J Trop Med Hyg. (2018) 98:248–51. 10.4269/ajtmh.17-0392 29141745 PMC5928713

[13] Mariotto AB Rowland JH Ries LA Scoppa R Feuer EJ. Multiple cancer prevalence: a growing challenge in long-term survivorship. Cancer Epidemiol Biomarkers Prev. (2007) 16:566–71. 10.1158/1055-9965.EPI-06-0782 17372253

[14] Lucchinetti CF Kimmel DW Lennon VA. Paraneoplastic and oncologic profiles of patients seropositive for type 1 antineuronal nuclear autoantibodies. Neurology. (1998) 50:652–7. 10.1212/WNL.50.3.652 9521251

[15] Tschernatsch M Dierkes C Gerriets T Hosch J Stolz E Kaps M. Paraneoplastic neurological syndromes in patients with carcinoid. Eur J Neurol. (2008) 15:1390–4. 10.1111/j.1468-1331.2008.02328.x 19049559

